# Review on 3D printing techniques for cutting tools with cooling channels

**DOI:** 10.1016/j.heliyon.2023.e22557

**Published:** 2023-11-19

**Authors:** Anuj Srivathsa S S, Muralidharan B

**Affiliations:** Department of Manufacturing, School of Mechanical Engineering, Vellore Institute of Technology, Vellore, India, 632014

**Keywords:** Additive manufacturing, Cutting tools, Advanced tooling, High-speed machining

## Abstract

This review paper critically emphasizes the possibilities and recent developments for producing high-performance conventional cutting tools that are in demand in the traditional machining industry. These cutting tools are considered for use in traditional machining of materials that provide a high strength-to-weight ratio for various applications with high precision. This review focuses on the machinability of turning, milling, drilling, and other special tools manufactured using various additive manufacturing methods. The materials and methods used are also studied, enabling us to understand the vast requirements of tool materials and the additive manufacturing methods available for production. The critical suggestions discussed would establish a platform for the selection of printing methods and printing strategies to develop cutting tools with complex internal geometries.

## 1 Introduction

**Fig. 1:**
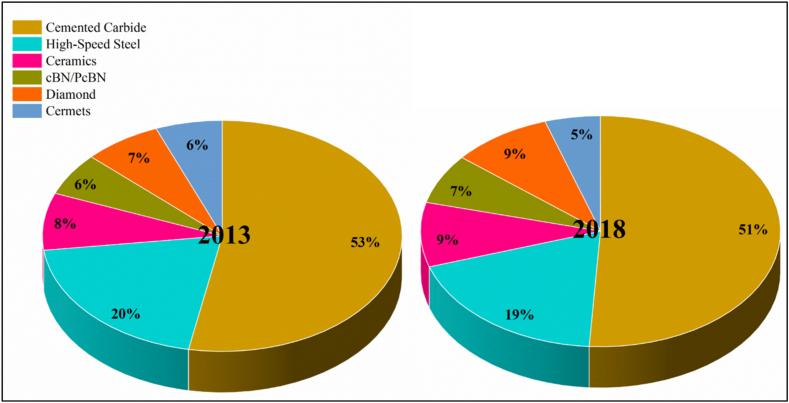
Turning tools materials distribution chart in industries for 2013 and 2018.

Looking in the context of developing new materials that have enhanced characteristics for better performance than existing materials is desired to improve the product life and ultimately reduce the cost of production. Currently, the cost of cutting tools used in the manufacturing sector is a major factor that determines the overall cost of manufacturing and, ultimately, the product's final price. These cutting tools are used to machine high-value components such as turbine blades, machine parts, automotive and aerospace parts, and biomedical implants. The properties of the materials used for making these products are superior to those of the rest, where the thermal conductivity is very low and the strength is very high. The main focus of today's manufacturing research is to improve productivity; thus, the focus is more on improving cooling and lubrication that can be achieved during the machining of these latest materials with enhanced strength [1]. According to the recent trend in industries, more than 80 % is still a top-down process, with machining dominating as several processes, such as turning, milling, drilling, etc., are used to achieve the desired final output. According to a research survey conducted by Atiqah [2] and Rizzo [1], the trends in cutting tool materials are shown in Fig. 1.

Fig. 1 shows that in 2013, the percentage usage of the most common cutting tool materials was cemented carbides (53 %) and high-speed steels (HSS) (20 %), and the rest were ceramics, cBN/PcBN, diamond and cermets. The same data, when seen in 2018, reveals that the usage of cemented carbides has reduced slightly to 51 % (a 2 % decrease), whereas the usage of HSS has reduced by only 1 %–19 % in total. This survey data shows the importance of small-scale industries, as the usage of HSS is dominant in this sector. The main reasons for the constant usage of HSS in the machining industry are that they are cheaper, easier to use and can be reused after a minor rework. Therefore, justifiable research for improving the tool life of HSS and other materials is needed [[3], [4], [5], [6], [7], [8]].

Cutting fluid usage for machining is well-known for removing high temperatures from the cutting zone and lubricating the tool-chip interface and workpiece contact zone to reduce wear. However, cutting liquids is deemed harmful to the operator and the environment. Thus, the alternatives for flooding methods, for example, minimum quantity lubrication (MQL) and other subsidiary methods of cooling techniques, are developed and implemented as much as possible. This indirect and other method development has paved the way for greener and more sustainable machining [[3], [4], [5], [6], [7], [8]]. According to previous literature, the critical factor in defining a method as green or sustainable is when the cutting fluid usage is significantly reduced. This reduction can be achieved by any means, including reduced quantity, increased reuse, reduced cost, improved performance by additives, and various other such methods. The materials of interest in the machining industries to produce the above-mentioned high-priority parts are superalloys [8], Al-7075 alloys [9], titanium alloys [10,11], Inconel alloys [12], various carbon steels [6], high-entropy alloys [13], and various other metal matrix composites [7], such as SiCP/Al [14] and Al–12Si [15].

Since the 1980s, different additive manufacturing technologies have emerged, permitting the usage of an extensive variety of materials. Various materials such as Al and Mg alloys, Ti alloys, various grades of steels, Ni and Co-based superalloys, intermetallic compounds, Al matrix, Ti matrix, Ni matrix, Co matrix, Fe matric compounds, ceramics, gradient materials, biomaterials, and even high entropy alloys can be produced efficiently via additive manufacturing methods [[16], [17], [18], [19]]. Reducing the time required for developing, commercialising, and producing parts is needed to produce components in industries economically. Additive manufacturing fulfils these requirements, enabling manufacturers to survive the competitive market. Using 3D printing technology, fabricating tools with geometries that are not achievable using conventional methods is facilitated [20]. The need for special tools for machining, especially in hard-to-cut and highly heat-resistant materials such as tungsten, nickel-based alloys, and unique materials such as shape memory alloys, is emerging. During the machining of these materials, the heat transfer from the workpiece to the cutting tool is very high because the thermal conductivity of tool materials is very high, and that of the workpiece is very low. Therefore, the cutting tool material induced thermal stress due to high heat flux. The primary cause of tool wear mechanisms found during the machining of these materials is the temperature at the cutting zone, which is the primary factor for the wear. Thus, the thermal stress induced when machining parts made of these materials will increase the tool's wear, making it difficult to machine without proper cooling [[21], [22], [23], [24], [25], [26]]. A directed cutting liquid supply can meaningfully reduce this thermo-mechanically induced wear during any machining process used for producing parts through conventional machining processes. Many conventional methods for making internally cooled cutting tools have been performed earlier, which had numerous limitations in design and performance. In an experiment by Isik [27], an internally cooled cutting tool holder was intended and fabricated to aid green machining. In an experiment by Gupta [21], a sectional green-circuited internal cooling turning tool was designed, fabricated, and experimentally verified for its cutting performance. In an experiment by Li et al. [28], a novel internally cooled turning tool was designed and experimentally verified for its performance. Here, various topological designs were made, which would cool the insert from the bottom and increase the tool's life.

In an experiment by Ozturk et al. [29], a modified cutting holder with a seat for cutting inserts was designed to provide internal cooling to the lower surface of the insert. When using this tool for a machining operation, the temperature reduction noted was 106 °C only when cooled cutting fluid was used. This level of temperature reduction is achieved when a cooled cutting fluid (by direct cooling or by using cryogenic cooling systems) is used as the working fluid, and this level of cooling cannot be achieved when using an average room temperature working fluid. A novel internal cooling and spraying tool was manufactured through a conventional machining process and assembled according to the design needs in an experiment conducted by Shu et al. [30]. This novel composite cutting tool exhibits significantly reduced temperatures and tool wear when used for a machining operation. In an experiment by Mohapatra et al. [22], a single-point turning tool was developed from scrap tools via centrifugal casting. This method facilitates recycling waste-cutting tools that become obsolete after their life cycle.

Likewise, many advanced cooling methods for specific machining operations have been experimented with by various researchers such as Ozturk [31] for internally cooled innovative cutting tools, John [32] for advances in micro cutting, Umair [33] for zig-zag milling using different cooling methods, Yao [34] for independent cooling to enhance dry cutting, Manuel [35] for internal cooling of diamond cutting tools, Liu [36] for internal spraying cooling for drilling and other such internal machining operations, Jose [37] for grooving to enhance cooling, and Luo [38] for cooling of tools for bone drilling.

Walder et al. [39] created micro holes using a laser drilling operation to create internal channels for internally cooled cutting tools. Here, the weakening due to thermal impact is reduced with the help of these cooling channels. A hole of 0.3 mm diameter cooling channel was created in a 2.5 mm diameter milling tool. In an experiment by Yin et al. [40], the cavitation occurring during a high-speed milling process was studied for the internal cooling process. From the simulation and experimental results, it was concluded that during internal cooling in high-speed milling, fish skin-like cavity pits and pinholes were formed on the flank face of the milling tool. In an experiment by Muller et al. [41], a study on the internal cooling of drilling was conducted to improve the fluid flow to critical areas during the drilling process. The CFD simulations concluded that structuring the faces will improve the fluid flow conditions to the critical region on the tool and workpiece interface. When using this tool for a machining operation, there was a significant upgrade in the life and workpiece surface quality. However, traditional manufacturing methods restrict these processes in design and performance; additive manufacturing methods can aid the freedom of complex designs.

Many direct and indirect ways of using additive manufacturing to produce tools are being researched and implemented successfully. Ghani et al. [42] created a turning tool holder with internal cooling channels using selective laser melting and metal laser sintering. The processing capabilities for producing cutting tools were studied by measuring the dimensional accuracy of the 3D printed part. The dimensional deviations were minimal in SLM and were the best among the two methods. Sergey et al. [43] studied the feasibility of producing different cutting tools concerning product design and incorporating internal cooling channels. Cost studies for various tools were also calculated for economical production. Using additive manufacturing methods, Jindrich et al. [44] produced indexable inserts with internal cooling channels in the design. Design optimization was performed to increase the fluid flow through the cooling tracks produced via the additive manufacturing technique and compared with conventionally made holes. Complex-shaped cutting inserts made up of zirconia by Rongxuan et al. and alumina by Maopeng et al. were experimentally investigated, as reviewed by Yang [45], which gives us much-needed insight into the production of ceramic cutting tools. The experimental investigations they performed concluded that these tools could be used in harsh cutting conditions such as high temperatures, highly corrosive environments, etc.

The indirect additive manufacturing method for depositing W–C–Co on a stainless steel substrate was performed by Amit et al. via directed energy deposition (DED) [46]. The deposited material was ground into a turning tool using a tool and cutter grinding machine and was further used for machining operations. The results showed that tool life increased when machining hard materials. Polymeric moulding casts were produced via the stereolithography (SLA) method and the material extrusion indirect additive manufacturing process by Kim et al. as stated in the review by Yang [45]. In those as mentioned earlier indirect additive manufacturing methods, W–C–Co cutting inserts were produced and characterized for their mechanical properties. The casting of W–C–Co is challenging because of its high melting temperature; thus, moulds made by additive manufacturing will aid in the powder metallurgical process of producing inserts.

Michal et al. [20] experimentally studied using AISI 1.2709 steel for producing milling tools via the SLM technique. When analyzing the dimensional accuracies and the surface finish of the as-printed parts, it was found that the SLM process needed a 5 % allowance to achieve higher dimensional accuracies. Milling tools produced by Lakner et al. [47] using AISI 1.2709 material were compared with conventional tools. The additive manufacturing method tool could incorporate a complex internal cooling channel due to the design freedom, improving machining performance. The internal cooling channel designs for additive manufacturing were optimized in the test piloted by Zachert et al. [48]. Three different geometries were designed, and the flow characteristics were studied. The demonstration for producing a drilling tool using M2-grade high-speed steel using SLM was performed by Sander et al. [49]. Process parameters were optimized to produce dense and crack-free parts. This review paper emphasizes the importance of making conventional cutting tools via additive manufacturing methods with specific design advantages.

### 1.1 State of the art in machining

In this chapter, the state of the art in various aspects of machining will be discussed in brief, which enables us to understand the technical aspects in a better way. The various aspects include temperature, tool wear, tool life, cutting forces, chatter, tool stability, surface roughness, precision, dimensional accuracy and other such details associated with machining various metals and alloys for numerous day-to-day applications.

Thin-walled parts have become a significant attraction for numerous applications because the means for producing meaningful thin-walled features have advanced exponentially over the past decade. When considering the machining of these thin-walled parts, a major concern of chatter in the tool must be addressed [50]. The dynamics aspect of machining in addressing chatter and its subsequent reactions, such as reduced surface quality and machining efficiency, is needed when considering the machining of thin-walled parts. Research for chatter elimination of suppression is needed, and it can be done by varying the cutting tools' designs, which can benefit from the AM of tools. When machining super alloys [6,[8], [9], [10], [11], [12], [13], [14], [15]] is concerned, the conventional flooding method cannot be considered the most efficient as the economic and sustainability aspects of machining do not allow it to be the best. However, it provides better performance in cooling. The growth of the machining industry and its contribution to global economic growth is due to the linear production it offers. Due to this reason, the consumption of resources for achieving the demand in quality and quantity cannot be maintained over a long period. Thus, the paradigm shift towards greener and sustainable utilisation of resources needed for cooling and lubrication is needed to fulfil the manufacturing needs, hazards related to occupation, and legislation requirements focused on in the recent past [[4], [5], [6], [7], [8]]. From various previous studies [[3], [4], [5], [6], [7], [8], [9], [10], [11], [12], [13], [14], [15]], it is evident that the maximum temperature reduction in conventional methods of cooling and lubrication is in the range of 100–150 °C, the cutting force reduction is in the 25–30 %, the reduction in tool wear via enhanced lubrication is only 30–50 μm, the achieved surface roughness is above 1 μm with a minimal increase in tool's life. The scope for reduction of these parameters is high as the need for producing parts with high quality and quantity is always increasing. This requirement can be fulfilled by adapting hybrid cooling methods.

### 1.2 State of the art in cooling methods

In an experiment by Isik [27], an internally cooled tool was used to machinate nickel-based superalloys. Here, fluid flow holes of 2 mm diameter with openings for entry and exit were fabricated and experimentally tested with cooled water as the working fluid for machining. The results show that when using this novel turning tool, the maximum temperature at the tip throughout machining was reduced by 9 %, and the tool life was extended by 12 % compared with dry machining. In an experiment by Tao et al. [51], a circular internally cooled turning tool holder was designed in which the cutting fluid enters the chamber from the bottom face of the tool and exits via the tool's back end. From the results of the experimental verification of tool tip temperature, the temperature when using this novel internal turning tool was 336.3 K. There was a 67.9 °C reduction in temperature when compared with dry machining.

Li et al. [28] developed an internally cooled turning tool with adaptors for inserts, optimized topological channels for effectively cooling the inserts and fabricated it via a conventional machining process. From the turning experimental results, we can conclude that the optimized internally cooled cutting tool performed better as there was a 16 °C reduction in temperature at the tip. In an experiment by Shu et al. [30], a combined circular and spraying internally cooled turning tool was established to enhance the cutting performance. CFD analysis was performed to optimize the coolant channel. The practicality experiment found that the tool would perform better compared with conventional tools available in the market.

Yin et al. [40] studied the cavitation occurring during a high-speed milling process for the internal cooling process. Cavitation is the pressure difference between the flank and rake faces during high-speed tool rotation. The low-pressure zone created on the flank face due to cavitation causes damage to both the workpiece and cutting tool, resulting in increased surface roughness in the workpiece and premature loss of the cutting edge in the tool. In an experiment by Muller et al. [41], various internal cooling designs and face structuring were designed and simulated to improve cutting fluid flow to the critical region to enhance drilling performance. From the results, we can see that structuring the drilling tool improved the drilling performance significantly when machining tough-to-cut materials such as Inconel-718. The improved cutting fluid conditions improved tool life by 22 % when experimentally verified.

Researchers Ozturk et al. [31] designed a novel tool to fit well within green manufacturing and eco-friendliness because fluid usage is shallow. The designed smart, internally cooled cutting tools measure the temperature of the cutting fluid used at the inlet and outlet points, which is used to study the measure of heat removed from the cutting zone. The developed ICSCT system consists of a circular cutting fluid system where the hot working fluid from the outlet is cooled with an intelligent system consisting of a radiator setup. John et al. [32] studied the need for internal cooling methodologies and explored their critical advantages. Umair et al. [33] investigated zig-zag milling using different cooling methods, emphasizing the need for cooling at the tool tip during freeform machining. Yao et al. [34] investigated independent cooling methods to enhance dry cutting with an advanced cooling chamber placed below the insert. This would also enhance the instrumenting for measuring the temperature at the tooltip. Manuel et al. [35] investigated internal cooling for diamond cutting tools, as diamond tools can be used for high-hardness materials. The novel cooling created here enhanced the cutting performance by 20 %. Liu et al. [36] investigated an internal spraying cooling method for drilling and other such internal machining operations for machining high-hardness materials, and it was found that the optimized tool produced a better surface finish than the conventional tool because of the enhanced cooling achieved by spray cooling. Luo et al. [38] investigated the cooling of tools for bone drilling applications because cooling and burr removal are very essential in medicine and dentistry.

The research works shown in Table 1 emphasize the need for unique cooling systems for the machining industries to economically machine high-hardness materials such as titanium and nickel-based superalloys. It should also be noted that almost every unique tool was made via a conventional machining process, and therefore, the designs were limited. This limitation in design had an adverse effect on its functionalities and interfered with cutting performance, which is the main objective of the special tools.

**Table 1:** State-of-the-art in advanced internally cooled cutting tools.

S. No	Authors	Tool	Type of Cooling	Significant Outcome
Turning Tools				
1	Isik [27]		Circular MQL	Tool life improved by 12 %
4	Shu et al. [30]		Circular and Internal MQL	A 38 °C reduction in max temperature
5	Yao et al. [34]		Circular MQL	30 % reduction in cutting temperature
**Milling Tool**				
6	Walder et al. [39]		Internal MQL	Laser drilling in milling tool for MQL channels
**Drilling Tools**				
7	Muller et al. [41]		Internal MQL	Tool life improved by 22 %
8	Liu et al. [36]		Internal Spray cooling	41–44 % reduction in drilling temperature and 9.93 % reduction in surface roughness
9	Manuel et al. [35]		Internal Cooling	50 % reduction in flank wear

### 1.3 Additive manufacturing of cutting tool

#### 1.3.1 Turning tools

**Fig. 2:**
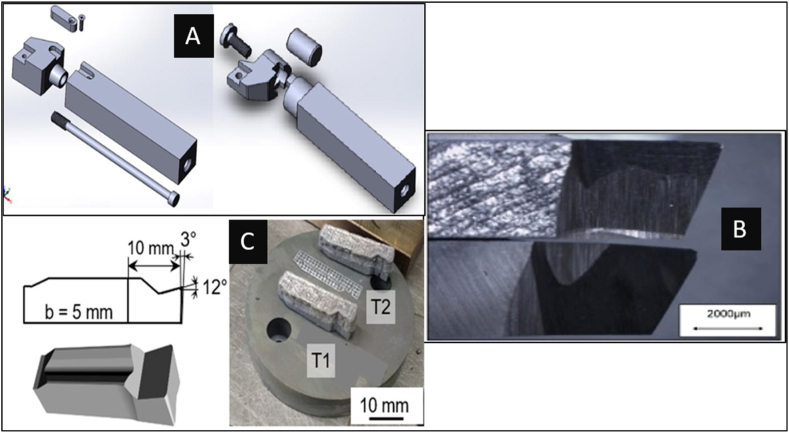
Turning tools produced via additive manufacturing methods (A- [43]; B- [46]; C- [54]).

Ghani et al. [42] experimentally compared the dimensional accuracies of internally cooled cutting tool holders 3D printed using powder bed fusion (SLM and DMLS) processes. The internal channel's inlet, outlet, and channel diameters and surface roughness were measured using destructive methods. The standard error obtained was 5.12 % for SLM and 2.88 % for DMLS. The SLM process produced a better surface finish than DMLS, thus concluding that the SLM process performed better, although it produced a higher error percentage. The possibility and the effectiveness of using additive manufacturing methods for producing conventional cutting tools were analyzed by Sergey et al. [43]. Here, a hybrid method for implementing additive manufacturing methods was suggested, where the shank was designed to be made conventionally, and the replaceable head's designs were made so that it could be additively manufactured, as shown in Fig. 2a The tool head design incorporated a coolant flow channel to enhance the cutting performance. It was predicted that the tools produced by this hybrid method could be at par with traditionally made tools in terms of strength, hardness, and other vital aspects such as the economy of production cost. These properties could be easily achieved due to the high density, surface roughness, and other mechanical properties that the SLM machines at the market could provide. The design and manufacturing of indexable inserts with internal cooling channels focused on enhancing the machining performance in an experiment conducted by Jindrich et al. [44]. Conventional manufacturing and additive manufacturing of internal cooling channel designs in tools and inserts were compared in this study. Optimized designs in shapes and sizes were proposed for increased jet velocity, which could be easily achieved via additive manufacturing techniques. The design aspects of producing cutting tools with AM for precise working fluid supply are impactful because only through design for AM the junction required for diverting the cutting fluid from the shank to the rake and flank faces can be made favourable without causing any hindrance to the intended purpose. Successful fabrication of complex-shaped zirconia cutting tools was performed using additive manufacturing stereolithography (SLA) in an experiment conducted by Rongxuan et al. as mentioned in the review done by Brooke [52] and Greta [53]. This tool had a withdrawal groove that would improve the tool's replacement time. The achieved density of 97.14 % and dimensional accuracies achieved with 35.26 % shrinkage concluded that highly dense and intricate geometry in the body of the insert, such as a honeycomb structure, could be easily incorporated and achieved through the SLA method.

A fully dense alumina cutting tool was fabricated using SLA in an experiment conducted by Maopeng et al. as mentioned in the review by Brooke [52] and Greta [53]. Different drying and debinding methods were tested to optimize the process and increase the density of the final part. A high density of 99.3 % and high hardness of 17.5 GPa was achieved in this study when optimized process parameters were used. It can be concluded from the high hardness achieved that using high-hardness ceramic tools would be very beneficial due to the insufficient properties of cutting tool materials in the machining industry. The significant benefits of ceramic tools are that they can function at very high temperatures and corrosive surroundings, and where good tribological, mechanical, and chemical resistance is needed.

In an experiment conducted by Amit et al., multi-layer deposition of W–Cr–Co (Stellite) was performed on a stainless-steel base workpiece via directed energy deposition (DED) [46]. The deposited specimen was then grounded into a turning tool, as shown in Fig. 2b, using which a machining experiment was conducted. The reciprocating wear test revealed 38 % less wear than the black alloy-525 superalloy. Constant and low rake and flank wear were observed while turning a stainless-steel workpiece. These results demonstrate the use of additive manufacturing to fabricate job-specific tools for advanced machining. The indirect method for fabricating W–C–Co cutting tools was reviewed by Yang et al. [45], and the study of Kim et al. was reviewed. This study used the SLA method to produce polymeric casting or powder metallurgy moulds with complex profiles to enhance cutting performance for turning inserts. Because casting the W–C–Co cemented carbide alloys is difficult due to a melting point of 2870 °C, they are usually produced via powder metallurgy methods. Here, 99 % density parts were made using the 3D-printed moulds, and the results validated the mechanical characteristics of this method for industrial-grade tools. Kim et al. also demonstrated the production of W–C–Co tools via the material extrusion indirect additive manufacturing process. W–C–Co slurry was prepared and 3D printed using a 0.4 mm diameter nozzle. High-dense parts (99.3 %) were manufactured, and no difference in mechanical properties was observed compared with conventionally made tools. This comparison study was conducted for additive manufactured and powder metallurgical W–C–Co parts with the same sintering conditions. It was concluded that this method would improve design freedom, production time, and economics. In an experiment performed by Sofia et al. [54], W–C–Co was 3D printed using powder-bed heating of 900 °C to produce indexable inserts (as shown in Fig. 2-C) for turning. Highly dense and defect-free inserts were successfully 3D printed with the achieved build platform temperature. The results showed that the tool wear using commercial WC and 3D printed inserts showed similar mechanisms in wear until its life.

A lower dimensional error percentage (2.88 %) and better surface finish (9.037 μm) were achieved by Ghani et al. [42] using the SLM technique. SLM is found to be the better method to produce metal turning tools/holders when the parameters are optimized. With the achievable design freedom, higher density of parts, and ease of post-processing techniques, it can be concluded that SLA is the best additive manufacturing method for producing ceramic cutting inserts. Novel monolithic turning tools with transpiration cooling channels were designed, 3D printed by Anuj et al.[55] and were studied for its dimensional accuracy. From this experiment it is evident that a 5-10% allowance is required for creating transpiration cooling channels via PBF method. Direct cutting inserts (with materials like W–C–Co [[56], [57], [58], [59]] and ceramics) with a higher density of 99.9 % and 10 % higher hardness are manufactured using indirect processes such as DED, slurry extrusion, and mould production. However, achieving a stable cutting edge requires extensive post-processing (mechanical machining, drying, degreasing, and sintering). Due to this reason and the availability of the SLM technique to process W–C–Co when optimized, indirect methods are unsuitable for cutting tool/insert production.

#### 1.3.2 Milling tools

**Fig. 3:**
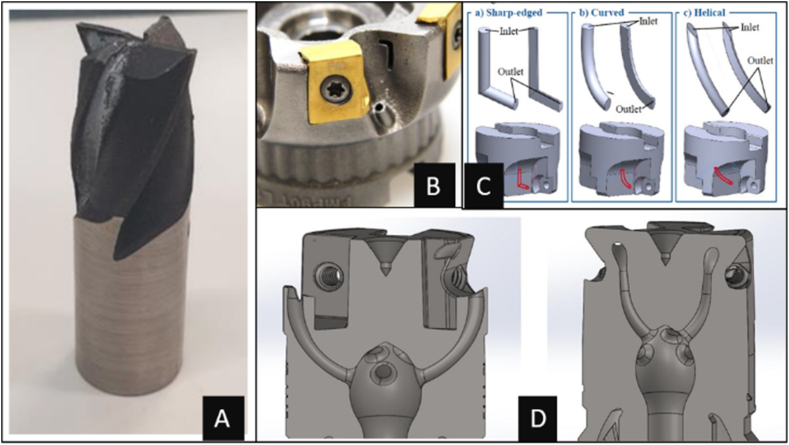
Milling tools produced via additive manufacturing methods (A- [20]; B- [47]; C- [48]; D- [43]).

Michal et al. conducted A study on maraging steel 1.2709 using SLM technology to produce an end milling tool [20]. The 3D-printed end miller cutter was examined for its dimensional accuracy and surface roughness. The critical parameters to be considered when designing end milling tools were cylindricity, length of the shank, and edge radius. Achieving the required cutting radius of 50 μm was complicated, and extensive post-processing, i.e., machining, was required. As shown in Fig. 3-a, the as-printed cutter needed machining as post-processing to achieve higher dimensional accuracy and obtain sharp cutting edges. An allowance of 0.5 mm was required when designing to provide for this post-processing. An experimental comparison of the performance of the AISI-1.2709 milling tool with a focused cutting fluid supply produced via conventional and additive manufacturing methods was made by Lakner et al. [47]. The influence of coolant nozzle designs and their placement on the tool holder, as shown in Fig. 3-b, on tool wear, was the objective of the investigation. The tool wear for the additive manufactured tool was lower, reaching 67 % more tool travel length. The L-shaped nozzles provided good lubrication to the tool-chip interface. When comparing the machining results of the additive-manufactured tool holder and the conventionally made tool holder, there were no significant deviations in cutting forces. However, the tool wear rate reduced significantly in favour of the additive manufactured tool, thus concluding that the AMed milling tool can be used for machining purposes to fulfil the industrial needs to produce parts with high quality at a better economy. The additive manufacturing method can also produce complex designs such as L-shaped nozzles. In the trial piloted by Zachert et al. the optimization of internal channel designs was performed [48]. Three different L-channels, sharp-edged, curved, and helical geometries, were designed, as shown in Fig. 3-c. The studies were conducted to determine the strength of the part and the volumetric flow rate of the cutting fluid to enhance the cutting performance of the milling tool holder. Due to the reduced internal friction and sharp edges in the 3D printed internal fluid flow channels, the flow rate of additive manufactured holes increased by 23 % compared to conventionally drilled holes, which would result in sharp edges and high internal surface roughness.

The feasibility of producing complicated internal channel designs using a milling tool was studied by Sergey et al. [43]. It was concluded that creating conical channels-holes with a curvilinear axis, was possible only through additive manufacturing methods, as shown in Fig. 3-d, as conventional methods, could only make straight holes. Here, similar opportunities for improving the designs for hull end milling tools are also studied, where designs to enhance the performance of the milling tools will be very much feasible through additive manufacturing methods only. It was found that the feed was the highly significant factor, with a 91.57 % contribution. A survey was conducted by Pavel et al. to optimize the topology of a milling tool to enhance the weight-to-strength ratio [60]. According to significant indications from the survey results, incorporating various lattice structures into the tool's shank to reduce the functional weight of the part will be a potential innovation, allowing a higher degree of dynamics in cutting applications. The vast requirement for practical approaches to achieve lighter cutting tools, reducing vibrations, and balancing when using rounded inserts could be understood from this survey.

The SLM process's milling tool created using AISI 1.2709 achieved very high dimensional accuracy (99 %) and density (99.5 %). It only required minimal post-processing (machining) and a design allowance (5 %) to maintain the cutting edge. The design optimization is performed using CFD analysis for the flow characteristics of the internal cooling chambers. A 23 % increase in fluid flow through the internal channels built using the SLM technique was achieved. The optimized milling tools were printed using AISI 1.2709 material. When it was used for the machining experiment, there was a 67 % increase in the travel path, and the cutting forces were reduced by 2.5 %. The discussions above show that the SLM technique enables the production of milling tools with any material of interest.

#### 1.3.3 Drilling tool

**Fig. 4:**
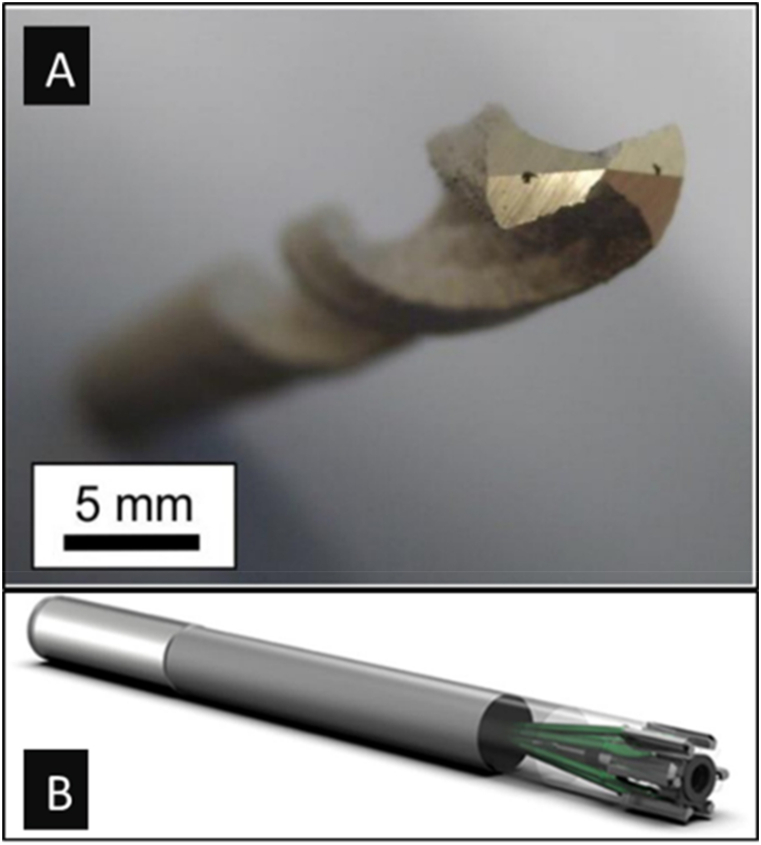
Drilling tools produced via additive manufacturing methods (a- [49]; b- [43]).

A successful demonstration was performed by Sander et al. for producing a drilling tool via SLM using M2-grade high-speed steel [49]. The SLM process parameters were optimized to make crack-free and dense parts. The SLM's localized heat input and sudden cooling resulted in a homogenously fine microstructure and mixed phases. These combined effects of different phases and fine microstructure resulted in outstanding mechanical properties such as micro-hardness of 900 ± 12 HV 0.1 and compressive strength of 3796 ± 163 MPa without any post-processing. In addition, a 6 mm drilling tool with internal cooling channels was printed to demonstrate the application of SLM in advanced tooling, as shown in Fig. 4-a. A feasibility study for a hybrid-designed drilling tool was done by Sergey et al. as shown in Fig. 4-b, where the body was designed to be made conventionally, and the replaceable head's design could be additively manufactured [43]. This method made numerous customization feasible, which would enhance the drilling performance. Design for additive manufacturing will also be economical in production via any additive manufacturing method, as the time taken and the cost for production will be significantly reduced.

#### 1.3.4 Other special-purpose tools

**Fig. 5:**
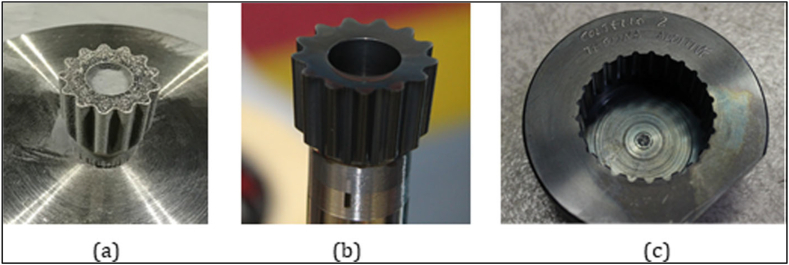
Special tools-gear scuddling tool [61] (a-as printed tool; b-after post processing; c-machined internal gear in workpiece).

A W–C–Co gear-scudding tool was manufactured via SLM in an experiment performed by Fortunato et al. [61]. Process parameter optimization and scan strategies were studied to improve the surface finish and dimensional accuracy of parts relating to the chemical composition of the feedstock used. The accuracy was high when the additive-manufactured internal gear scudding tool, as shown in Fig. 5, was used to produce internal gears for automotive applications, and the tool wear rate was minimal. In addition, the tool maintained its cutting edge without chipping during the machining operation because of the effects of the post-processing techniques incorporated here. There was a larger distance between the rake and clearance faces from 11 μm to 21 μm. In an experiment conducted by Douglas et al. excavation tools for extra-terrestrial applications were manufactured via SLM using multiple materials such as titanium alloy, stainless steel, tool steels, and maraging steel [62]. The tools were tested for properties and morphologies to replicate unknown territories and harsh temperatures. The increased hardness of the as-printed tools enhanced the cutting performance required for extra-terrestrial applications.

Table 2 is a collection of the process parameters used for optimized printing different cutting tools using various additive manufacturing methods in different machines. From this table, we know the availability of various additive manufacturing methods for 3D printing various materials according to the need and interest with optimized process parameters to achieve defect-free parts with the highest density and mechanical properties at par with conventional production methods.

**Table 2:** Various process parameters used for materials for producing cutting tools.

S. No	Authors	Methods	Process Parameters	Outcome
Turning Tools				
1	Ghani et al. [42]	SLM and DMLS	**SLM**: Power (W) 350; Hatching spacing (mm) 0.17; Scanning speed (mm/s) 930; Layer thickness (μm) 50.	The standard error obtained was 5.12 % for SLM and 2.88 % for DMLS
			**DMLS**: Power (W) 195; Hatching spacing (μm) 0.1; Scanning speed (mm/s) 800; Layer thickness (μm) 20.	
2	Amit et al. [46]	DED	Laser Power (W) = 410; Hatch Spacing (mm) = 0.5; Scan Speed (mm/s) = 5.5; Layer Thickness (um) = 500; Effective Energy Density (J/mm3) = 300.	Reciprocating wear test revealed 38 % less wear
**Milling Tools**				
3	Michal et al. [20]	DMLS	Laser Power (W) = 200; Scan Speed (m/s) = 4.5; Focus Diameter (um) = 50; Layer Thickness (um) = 20.	Achieving the required cutting edge radius of 50 μm was difficult, and extensive post-processing was needed
**Drilling Tool**				
4	Sander et al. [49]	SLM	Laser power (W) = 175; Hatching spacing (μm) = 60; Scan speed (mm/s) = 450. Laser power (W) = 125; Hatching spacing (μm) = 70; Scan speed (mm/s) = 400.	The SLM samples show superior properties without post-processing, compression strength of 3796 ± 163 MPa and microhardness of 900 ± 12 HV 0.1
**Other Special Tools**				
5	Fortunato et al. [61]	SLM	Laser Power (W) = 150; Focus Diameter (um) = 50; Layer Thickness (um) = 20 and 60.	Tool did not report chipping, other macroscopic alterations to the geometry after machining

## 2 Conclusion and future directions

This review paper explored additive manufacturing processes for fabricating different cutting tools. Specifically, the production of various cutting tools for turning, milling, drilling, and other operations.

• The thermo-mechanically induced wear on the tool in any cutting operation can be reduced by providing cutting fluid to the cutting zone internally, which enhances cutting performance. These intricate internal and external geometries can be produced using additive manufacturing techniques.
• So far, cutting tools have been produced via the indirect additive manufacturing method, where a cutting edge is achieved only by incorporating post-processing techniques. These production methods diverge from additive manufacturing's advantages in all aspects, from time to economic production of functional parts.
• Metals such as AISI 1.2709, Stellite, and ceramics such as zirconia and alumina are used for producing cutting tools that can only be used for a particular application. With the development and growth of the additive manufacturing sector, all tool materials that can be directly used as cutting tools, such as high-speed steels and WC, can be 3D-printed efficiently.
• Additive manufacturing techniques can be employed to incorporate transpiration cooling, internal minimum quantity lubrication, and lattice structures with enhanced cutting performance.

## CRediT authorship contribution statement

**Anuj Srivathsa S S:** Formal analysis, Methodology, Writing – original draft. **Muralidharan B:** Conceptualization, Formal analysis, Investigation, Supervision, Writing – review & editing.

## Declaration of competing interest

The authors declare that they have no known competing financial interests or personal relationships that could have appeared to influence the work reported in this paper.
